# Bis(1-{bis­[2-(diphenyl­phosphino­yl)eth­yl]phosphan­yl}-2-(diphenyl­phosphan­yl)ethane)dinitratoplatinum(II) methanol tetra­solvate

**DOI:** 10.1107/S160053680801533X

**Published:** 2008-06-07

**Authors:** Timo Paul Rieckborn, Emine Karakoc, Marc Heinrich Prosenc

**Affiliations:** a Institut für Anorganische und Angewandte Chemie, Department Chemie der Universität Hamburg, Martin-Luther-King-Platz 6, D-20146 Hamburg, Germany

## Abstract

In the title compound, [Pt(NO_3_)_2_(C_42_H_42_O_2_P_4_)_2_]·4CH_3_OH, the Pt atom positioned on a crystallographic centre of inversion. The two symmetry-equivalent nitrate anions are weakly coordinated to the Pt^II^ ion, creating, together with four P ligand atoms, a distorted octa­hedral coordination environment. In addition, several close C—H⋯O contacts between the nitrate O atoms and phenyl H atoms are found. Hydrogen bonds from two methanol solvent mol­ecules to one of the O—P groups complete the crystal structure.

## Related literature

For related literature on Pt^II^ complexes, see: Brüggeller *et al.* (1992 ▶). For a structure of a related Pt^II^ nitrato complex, see: Fernandez *et al.* (2001 ▶).
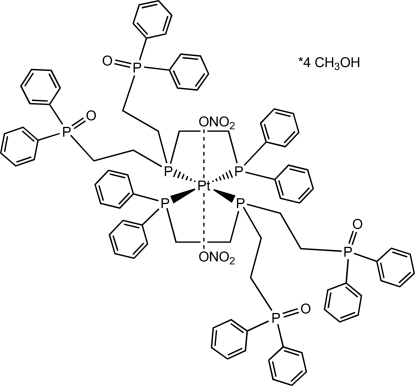


## Experimental

### 

#### Crystal data

[Pt(NO_3_)_2_(C_42_H_42_O_2_P_4_)_2_]·4CH_4_O
*M* *_r_* = 1852.64
Triclinic, 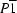

*a* = 9.9905 (9) Å
*b* = 14.3346 (13) Å
*c* = 16.9916 (15) Å
α = 66.200 (1)°
β = 77.479 (1)°
γ = 88.477 (1)°
*V* = 2168.4 (3) Å^3^
*Z* = 1
Mo *K*α radiation
μ = 1.83 mm^−1^
*T* = 153 (2) K
0.48 × 0.10 × 0.03 mm

#### Data collection

Bruker SMART APEX CCD area-detector diffractometer
Absorption correction: multi-scan (*SADABS*; Sheldrick, 1995 ▶) *T*_min_ = 0.474, *T*_max_ = 0.947
25181 measured reflections
9347 independent reflections
8160 reflections with *I* > 2σ(*I*)
*R*_int_ = 0.052

#### Refinement

*R*[*F* ^2^ > 2σ(*F* ^2^)] = 0.039
*wR*(*F* ^2^) = 0.068
*S* = 0.91
9347 reflections
512 parameters
H-atom parameters constrained
Δρ_max_ = 1.31 e Å^−3^
Δρ_min_ = −1.37 e Å^−3^

### 

Data collection: *SMART* (Bruker, 2000 ▶); cell refinement: *SAINT* (Bruker, 2000 ▶); data reduction: *SAINT*; program(s) used to solve structure: *SHELXS97* (Sheldrick, 2008 ▶); program(s) used to refine structure: *SHELXL97* (Sheldrick, 2008 ▶); molecular graphics: *SHELXTL* (Sheldrick, 2008 ▶); software used to prepare material for publication: *SHELXTL*.

## Supplementary Material

Crystal structure: contains datablocks I, global. DOI: 10.1107/S160053680801533X/si2089sup1.cif

Structure factors: contains datablocks I. DOI: 10.1107/S160053680801533X/si2089Isup2.hkl

Additional supplementary materials:  crystallographic information; 3D view; checkCIF report

## Figures and Tables

**Table:** 

Pt1—P2	2.3236 (9)
Pt1—P1	2.3399 (9)
Pt1—O4	3.524 (3)

**Table:** 

P2—Pt1—P1	83.40 (3)
P2—Pt1—O4	99.77 (5)
P1—Pt1—O4	104.36 (5)

**Table 2:** Hydrogen-bond geometry (Å, °)

*D*—H⋯*A*	*D*—H	H⋯*A*	*D*⋯*A*	*D*—H⋯*A*
O6—H6*C*⋯O2	0.84	2.32	3.160 (9)	174
O7—H7*A*⋯O2	0.84	1.95	2.777 (4)	170
C12—H12*A*⋯O4	0.95	2.29	3.132 (4)	147
C6—H6*B*⋯O4	0.99	2.53	3.294 (4)	134
C1—H1*B*⋯O5^i^	0.99	2.42	3.289 (5)	146
C1—H1*C*⋯O5^ii^	0.99	2.44	3.399 (5)	162
C8—H8*A*⋯O3^i^	0.95	2.58	3.376 (5)	142
C14—H14*A*⋯O4^ii^	0.95	2.56	3.317 (5)	137
C27—H27*A*⋯O6^iii^	0.95	2.50	3.338 (11)	147
C28—H28*A*⋯O2^iii^	0.95	2.60	3.385 (5)	141

Symmetry codes: (i) 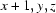; (ii) 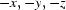; (iii) 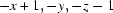.

**Fig. 1.**
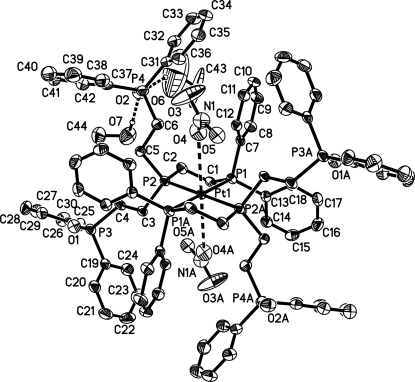
ORTEP representation of the PtII complex with displacement ellipsoids at 50% probability. H atoms and labels for symmetry equivalent C atoms have been omitted for clarity.

## supplementary crystallographic information

### Comment

The title compound, [Pt(*L*)_2_]^2+^ (NO_3_^-^)_2_ (*L* = (P1,*P*1-bis(2-(diphenylphosphinoxid)ethyl)-P2,*P*2-diphenylethane -1,2-diphosphine), crystallizes as a packing of a discrete dication and two nitrate anions with the Pt-atom positioned on a centre of inversion. The two symmetry equivalent ligands molecules *L* are coordinated to the Pt-center by two phosphine donor atoms P1 and P2 with Pt—P distances (*d*(Pt—P1) = 2.3399 (9) Å, d(Pt—P2) = 2.3236 (9) Å) (Table 1) creating a square planar coordination environment in accord with previously reported Pt^II^ tetraphosphine complexes (Brüggeller *et al.*, 1992). The two additional phosphor oxide groups of the ligands are not coordinated to the metal centre in the solid state. A long distance of d(Pt1—O4) = 3.524 (3) Å, angles of P1—Pt—O4 of 104.36 (5) ° and P2—Pt—O4 of 99.77 (5) ° are indicative of a weak Pt—NO_3_^-^ interaction which is much weaker than a previously reported Pt^II^—NO_3_^-^ distance of 2.116 Å in a related complex (Fernandez *et al., *2001). The nitrate anion is packed within a cavity created by the coordinated phosphine ligand atoms above and due to symmetry below the square plane spanned by the four phosphor ligand atoms creating a distorted octahedral coordination environment around the Pt^II^ ion. In addition several close C—H···O contacts between 2.30 and 2.70 Å were found between *ortho*-phenyl protons and methylene protons with nitrate oxygen atoms. Two methanol molecules with O—H···O hydrogen bonds to one of the O—P groups (Table 2) complete the crystal packing. The second P—O1 fragment exhibits weak hydrogen bond interactions with d(O1—H17A) of 2.997 Å and d(O1—H18A) of 2.991 Å to one of the adjacent phenyl group hydrogen atoms.

### Experimental

All reactions were carried out under an atmosphere of dry nitrogen using standard Schlenk techniques. Solvents were dried and stored under nitrogen. Platinum(II)nitrate was purchased from ChemPur and Tris(2-(diphenylphosphino)ethyl)-phosphine was obtained from Acros Organics.

150 mg (0.47 mmol) Platinum(II)nitrate was dissolved in 50 ml water and 50 ml e thanol was added. 315 mg (0.47 mmol) Tris(2-(diphenylphosphino)ethyl)- phosphine (PP3) was dissolved in dichlormethane and added to the reaction mixture. The suspension was stirred at room temperature for 3 days. Afterwards the mixture was concentrated to small volume and a yellow solid precipitated. The precipitate was filtered and dried *in vacuo*. Single crystals were received by gas phase diffusion of diethyl ether into a solution of the yellow product in methanol.

### Refinement

All non-hydrogen atoms were refined anisotropically and H atoms were refined using riding constraints with C—H distances set to 0.95 Å for aromatic, to 0.99 Å for aliphatic, 0.98 Å for methanol C—H bonds and 0.84 Å for O—H bonds. *U*_iso_(H) values were set to 1.2 *U*_eq_ of the parent atom.

### Crystal data

**Table:**

[Pt(NO_3_)_2_(C_42_H_42_O_2_P_4_)_2_]·4CH_4_O	*Z* = 1
*M**_r_* = 1852.64	*F*_000_ = 952
Triclinic, *P*1	*D*_x_ = 1.419 Mg m^−^^3^
Hall symbol: -P 1	Mo *K*α radiation λ = 0.71073 Å
*a* = 9.9905 (9) Å	Cell parameters from 5288 reflections
*b* = 14.3346 (13) Å	θ = 4.9–49.4º
*c* = 16.9916 (15) Å	µ = 1.83 mm^−^^1^
α = 66.200 (1)º	*T* = 153 (2) K
β = 77.479 (1)º	Plate, colourless
γ = 88.477 (1)º	0.48 × 0.10 × 0.03 mm
*V* = 2168.4 (3) Å^3^	

### Data collection

**Table:**

Bruker SMART APEX CCD area-detector diffractometer	8160 reflections with *I* > 2σ(*I*)
Monochromator: graphite	*R*_int_ = 0.052
*T* = 153(2) K	θ_max_ = 27.0º
φ and ω scans	θ_min_ = 2.4º
Absorption correction: multi-scan(SADABS; Sheldrick, 1995)	*h* = −12→12
*T*_min_ = 0.474, *T*_max_ = 0.947	*k* = −18→18
25181 measured reflections	*l* = −21→21
9347 independent reflections	

### Refinement

**Table:**

Refinement on *F*^2^	Secondary atom site location: difference Fourier map
Least-squares matrix: full	Hydrogen site location: inferred from neighbouring sites
*R*[*F*^2^ > 2σ(*F*^2^)] = 0.039	H-atom parameters constrained
*wR*(*F*^2^) = 0.068	*w* = 1/[σ^2^(*F*_o_^2^) + (0.0185*P*)^2^] where *P* = (*F*_o_^2^ + 2*F*_c_^2^)/3
*S* = 0.91	(Δ/σ)_max_ < 0.001
9347 reflections	Δρ_max_ = 1.31 e Å^−^^3^
512 parameters	Δρ_min_ = −1.37 e Å^−^^3^
Primary atom site location: structure-invariant direct methods	Extinction correction: none

### Special details

**Table:**

Geometry. All e.s.d.'s (except the e.s.d. in the dihedral angle between two l.s. planes) are estimated using the full covariance matrix. The cell e.s.d.'s are taken into account individually in the estimation of e.s.d.'s in distances, angles and torsion angles; correlations between e.s.d.'s in cell parameters are only used when they are defined by crystal symmetry. An approximate (isotropic) treatment of cell e.s.d.'s is used for estimating e.s.d.'s involving l.s. planes.
Refinement. Refinement of *F*^2^ against ALL reflections. The weighted *R*-factor *wR* and goodness of fit *S* are based on *F*^2^, conventional *R*-factors *R* are based on *F*, with *F* set to zero for negative *F*^2^. The threshold expression of *F*^2^ > σ(*F*^2^) is used only for calculating *R*-factors(gt) *etc*. and is not relevant to the choice of reflections for refinement. *R*-factors based on *F*^2^ are statistically about twice as large as those based on *F*, and *R*- factors based on ALL data will be even larger.

### Fractional atomic coordinates and isotropic or equivalent isotropic displacement parameters (Å^2^)

**Table:**

	*x*	*y*	*z*	*U*_iso_*/*U*_eq_	
Pt1	0.0000	0.0000	0.0000	0.01742 (6)	
P1	0.18605 (9)	0.10153 (7)	−0.00768 (6)	0.0217 (2)	
P2	0.14224 (9)	−0.01106 (7)	−0.12330 (6)	0.0210 (2)	
P3	0.24812 (10)	−0.28401 (7)	−0.17502 (6)	0.0255 (2)	
P4	0.09161 (11)	0.23615 (7)	−0.36073 (6)	0.0293 (2)	
N1	−0.3437 (4)	0.2080 (3)	−0.0989 (3)	0.0416 (9)	
O1	0.1078 (2)	−0.29710 (18)	−0.18776 (16)	0.0335 (6)	
O2	0.2424 (3)	0.23539 (19)	−0.39496 (16)	0.0390 (7)	
O3	−0.3782 (3)	0.2601 (3)	−0.1679 (2)	0.1131 (17)	
O4	−0.2262 (3)	0.1769 (2)	−0.10285 (19)	0.0493 (8)	
O5	−0.4257 (3)	0.1826 (2)	−0.02587 (19)	0.0424 (7)	
C1	0.3381 (3)	0.0626 (3)	−0.0654 (2)	0.0253 (8)	
H1B	0.4176	0.1115	−0.0810	0.030*	
H1C	0.3608	−0.0060	−0.0269	0.030*	
C2	0.3073 (3)	0.0605 (3)	−0.1490 (2)	0.0266 (9)	
H2B	0.3817	0.0281	−0.1757	0.032*	
H2C	0.3040	0.1313	−0.1924	0.032*	
C3	0.1821 (3)	−0.1423 (2)	−0.1047 (2)	0.0237 (8)	
H3A	0.2208	−0.1713	−0.0515	0.028*	
H3B	0.0949	−0.1825	−0.0916	0.028*	
C4	0.2830 (4)	−0.1576 (2)	−0.1808 (2)	0.0271 (9)	
H4A	0.3787	−0.1501	−0.1760	0.032*	
H4B	0.2720	−0.1051	−0.2382	0.032*	
C5	0.0837 (4)	0.0428 (2)	−0.2265 (2)	0.0247 (8)	
H5A	0.1591	0.0444	−0.2757	0.030*	
H5B	0.0058	−0.0013	−0.2227	0.030*	
C6	0.0380 (4)	0.1520 (3)	−0.2456 (2)	0.0287 (9)	
H6A	0.0774	0.1798	−0.2102	0.034*	
H6B	−0.0634	0.1493	−0.2272	0.034*	
C7	0.1743 (4)	0.2356 (2)	−0.0763 (2)	0.0220 (8)	
C8	0.2892 (4)	0.3042 (3)	−0.1090 (2)	0.0308 (9)	
H8A	0.3748	0.2814	−0.0953	0.037*	
C9	0.2781 (4)	0.4063 (3)	−0.1616 (3)	0.0368 (10)	
H9A	0.3570	0.4529	−0.1854	0.044*	
C10	0.1528 (4)	0.4404 (3)	−0.1796 (2)	0.0373 (10)	
H10A	0.1454	0.5106	−0.2144	0.045*	
C11	0.0388 (4)	0.3729 (3)	−0.1472 (2)	0.0320 (9)	
H11A	−0.0471	0.3966	−0.1596	0.038*	
C12	0.0493 (4)	0.2698 (3)	−0.0964 (2)	0.0240 (8)	
H12A	−0.0289	0.2230	−0.0754	0.029*	
C13	0.2253 (3)	0.1005 (3)	0.0925 (2)	0.0233 (8)	
C14	0.2972 (4)	0.0223 (3)	0.1429 (2)	0.0299 (9)	
H14A	0.3304	−0.0294	0.1236	0.036*	
C15	0.3197 (4)	0.0204 (3)	0.2210 (2)	0.0347 (10)	
H15A	0.3700	−0.0322	0.2546	0.042*	
C16	0.2702 (4)	0.0938 (3)	0.2509 (3)	0.0396 (10)	
H16A	0.2847	0.0908	0.3053	0.048*	
C17	0.1997 (4)	0.1716 (3)	0.2014 (3)	0.0399 (11)	
H17A	0.1657	0.2223	0.2218	0.048*	
C18	0.1783 (4)	0.1762 (3)	0.1220 (2)	0.0308 (9)	
H18A	0.1317	0.2308	0.0875	0.037*	
C19	0.2724 (4)	−0.3735 (3)	−0.0693 (2)	0.0261 (9)	
C20	0.1804 (4)	−0.4595 (3)	−0.0227 (2)	0.0340 (10)	
H20A	0.1102	−0.4700	−0.0487	0.041*	
C21	0.1888 (5)	−0.5295 (3)	0.0600 (3)	0.0436 (11)	
H21A	0.1251	−0.5875	0.0908	0.052*	
C22	0.2916 (5)	−0.5138 (3)	0.0973 (3)	0.0495 (12)	
H22A	0.2984	−0.5618	0.1543	0.059*	
C23	0.3843 (5)	−0.4296 (3)	0.0531 (3)	0.0486 (12)	
H23A	0.4542	−0.4199	0.0797	0.058*	
C24	0.3753 (4)	−0.3594 (3)	−0.0300 (3)	0.0369 (10)	
H24A	0.4392	−0.3015	−0.0605	0.044*	
C25	0.3817 (4)	−0.2982 (2)	−0.2594 (2)	0.0240 (8)	
C26	0.3442 (4)	−0.3123 (3)	−0.3279 (2)	0.0337 (10)	
H26A	0.2501	−0.3134	−0.3301	0.040*	
C27	0.4446 (5)	−0.3247 (3)	−0.3938 (3)	0.0440 (11)	
H27A	0.4181	−0.3358	−0.4399	0.053*	
C28	0.5810 (5)	−0.3209 (3)	−0.3923 (3)	0.0444 (11)	
H28A	0.6490	−0.3287	−0.4374	0.053*	
C29	0.6188 (4)	−0.3057 (3)	−0.3250 (3)	0.0452 (11)	
H29A	0.7132	−0.3029	−0.3239	0.054*	
C30	0.5202 (4)	−0.2944 (3)	−0.2589 (3)	0.0382 (10)	
H30A	0.5476	−0.2840	−0.2128	0.046*	
C31	0.0423 (4)	0.3607 (3)	−0.3693 (2)	0.0321 (9)	
C32	0.1357 (5)	0.4441 (3)	−0.4216 (3)	0.0454 (12)	
H32A	0.2227	0.4346	−0.4524	0.054*	
C33	0.1019 (6)	0.5415 (3)	−0.4287 (3)	0.0630 (15)	
H33A	0.1659	0.5984	−0.4644	0.076*	
C34	−0.0245 (7)	0.5558 (4)	−0.3839 (3)	0.0664 (17)	
H34A	−0.0467	0.6224	−0.3885	0.080*	
C35	−0.1177 (5)	0.4744 (3)	−0.3330 (3)	0.0553 (14)	
H35A	−0.2049	0.4850	−0.3032	0.066*	
C36	−0.0856 (4)	0.3765 (3)	−0.3245 (3)	0.0426 (11)	
H36A	−0.1503	0.3202	−0.2883	0.051*	
C37	−0.0041 (4)	0.1944 (3)	−0.4207 (2)	0.0341 (10)	
C38	−0.1474 (5)	0.1935 (3)	−0.4052 (3)	0.0441 (11)	
H38A	−0.1969	0.2136	−0.3609	0.053*	
C39	−0.2181 (5)	0.1636 (3)	−0.4537 (3)	0.0600 (14)	
H39A	−0.3155	0.1630	−0.4424	0.072*	
C40	−0.1472 (7)	0.1346 (4)	−0.5184 (4)	0.0686 (16)	
H40A	−0.1956	0.1146	−0.5521	0.082*	
C41	−0.0054 (6)	0.1346 (3)	−0.5342 (3)	0.0628 (15)	
H41A	0.0434	0.1146	−0.5788	0.075*	
C42	0.0663 (5)	0.1637 (3)	−0.4850 (3)	0.0445 (11)	
H42A	0.1636	0.1625	−0.4956	0.053*	
C43	0.5176 (7)	0.3611 (8)	−0.3513 (5)	0.189 (5)	
H43A	0.4298	0.3719	−0.3181	0.283*	
H43B	0.5889	0.4101	−0.3563	0.283*	
H43C	0.5431	0.2914	−0.3204	0.283*	
O6	0.5062 (8)	0.3739 (8)	−0.4264 (6)	0.323 (5)	
H6C	0.4366	0.3392	−0.4223	0.484*	
O7	0.4255 (4)	0.0828 (3)	−0.3559 (2)	0.0696 (10)	
H7A	0.3781	0.1338	−0.3694	0.104*	
C44	0.4311 (5)	0.0373 (4)	−0.4169 (3)	0.0693 (15)	
H44A	0.4950	−0.0172	−0.4054	0.104*	
H44B	0.4629	0.0893	−0.4772	0.104*	
H44C	0.3393	0.0086	−0.4102	0.104*	

### Atomic displacement parameters (Å^2^)

**Table:**

	*U*^11^	*U*^22^	*U*^33^	*U*^12^	*U*^13^	*U*^23^
Pt1	0.01772 (11)	0.01445 (11)	0.02003 (12)	0.00337 (8)	−0.00161 (8)	−0.00841 (9)
P1	0.0203 (5)	0.0187 (5)	0.0269 (5)	0.0024 (4)	−0.0040 (4)	−0.0110 (4)
P2	0.0212 (5)	0.0184 (5)	0.0226 (5)	0.0036 (4)	−0.0014 (4)	−0.0094 (4)
P3	0.0274 (6)	0.0221 (5)	0.0281 (6)	0.0050 (4)	−0.0037 (5)	−0.0127 (5)
P4	0.0335 (6)	0.0252 (6)	0.0243 (6)	0.0036 (5)	−0.0035 (5)	−0.0067 (5)
N1	0.024 (2)	0.047 (2)	0.050 (3)	−0.0001 (18)	−0.0083 (19)	−0.016 (2)
O1	0.0253 (15)	0.0367 (16)	0.0403 (16)	0.0043 (12)	−0.0074 (13)	−0.0177 (14)
O2	0.0328 (16)	0.0393 (17)	0.0355 (16)	0.0016 (13)	0.0025 (13)	−0.0107 (14)
O3	0.044 (2)	0.175 (4)	0.052 (2)	0.013 (2)	−0.0171 (19)	0.025 (3)
O4	0.0298 (17)	0.055 (2)	0.065 (2)	0.0065 (15)	−0.0073 (16)	−0.0283 (17)
O5	0.0328 (17)	0.0443 (18)	0.0494 (19)	0.0025 (14)	−0.0046 (15)	−0.0208 (16)
C1	0.020 (2)	0.0203 (19)	0.035 (2)	0.0030 (16)	−0.0052 (17)	−0.0121 (18)
C2	0.022 (2)	0.025 (2)	0.032 (2)	0.0009 (16)	0.0017 (17)	−0.0147 (18)
C3	0.028 (2)	0.0185 (19)	0.025 (2)	0.0046 (16)	−0.0039 (17)	−0.0105 (17)
C4	0.029 (2)	0.0198 (19)	0.032 (2)	0.0054 (16)	−0.0019 (17)	−0.0122 (18)
C5	0.028 (2)	0.024 (2)	0.021 (2)	0.0027 (16)	−0.0019 (16)	−0.0094 (17)
C6	0.031 (2)	0.029 (2)	0.026 (2)	0.0117 (18)	−0.0061 (18)	−0.0121 (18)
C7	0.025 (2)	0.0199 (19)	0.023 (2)	0.0049 (16)	−0.0024 (16)	−0.0122 (16)
C8	0.028 (2)	0.026 (2)	0.037 (2)	0.0015 (18)	−0.0084 (19)	−0.0120 (19)
C9	0.039 (3)	0.024 (2)	0.043 (3)	−0.0067 (19)	−0.007 (2)	−0.010 (2)
C10	0.056 (3)	0.019 (2)	0.033 (2)	0.007 (2)	−0.012 (2)	−0.0071 (19)
C11	0.034 (2)	0.031 (2)	0.035 (2)	0.0125 (19)	−0.0123 (19)	−0.016 (2)
C12	0.023 (2)	0.023 (2)	0.025 (2)	0.0022 (16)	−0.0035 (16)	−0.0107 (17)
C13	0.019 (2)	0.025 (2)	0.026 (2)	−0.0022 (16)	−0.0032 (16)	−0.0118 (17)
C14	0.028 (2)	0.027 (2)	0.034 (2)	0.0050 (17)	−0.0048 (18)	−0.0131 (19)
C15	0.029 (2)	0.036 (2)	0.034 (2)	0.0038 (19)	−0.0110 (19)	−0.007 (2)
C16	0.046 (3)	0.045 (3)	0.033 (2)	0.000 (2)	−0.016 (2)	−0.017 (2)
C17	0.053 (3)	0.036 (2)	0.042 (3)	0.009 (2)	−0.018 (2)	−0.025 (2)
C18	0.035 (2)	0.028 (2)	0.033 (2)	0.0032 (18)	−0.0108 (19)	−0.0142 (19)
C19	0.031 (2)	0.022 (2)	0.026 (2)	0.0065 (17)	−0.0008 (17)	−0.0139 (18)
C20	0.040 (3)	0.028 (2)	0.033 (2)	0.0030 (19)	−0.002 (2)	−0.015 (2)
C21	0.060 (3)	0.024 (2)	0.037 (3)	0.005 (2)	0.001 (2)	−0.010 (2)
C22	0.068 (3)	0.035 (3)	0.037 (3)	0.024 (2)	−0.013 (3)	−0.005 (2)
C23	0.053 (3)	0.053 (3)	0.046 (3)	0.015 (3)	−0.023 (2)	−0.021 (3)
C24	0.039 (3)	0.034 (2)	0.038 (3)	0.005 (2)	−0.009 (2)	−0.014 (2)
C25	0.030 (2)	0.0147 (18)	0.024 (2)	0.0037 (16)	−0.0013 (17)	−0.0070 (16)
C26	0.038 (3)	0.032 (2)	0.032 (2)	0.0015 (19)	−0.007 (2)	−0.014 (2)
C27	0.055 (3)	0.051 (3)	0.030 (2)	0.000 (2)	−0.004 (2)	−0.022 (2)
C28	0.049 (3)	0.045 (3)	0.031 (3)	0.004 (2)	0.010 (2)	−0.018 (2)
C29	0.034 (3)	0.057 (3)	0.042 (3)	0.011 (2)	0.000 (2)	−0.022 (2)
C30	0.037 (3)	0.047 (3)	0.033 (2)	0.008 (2)	−0.004 (2)	−0.021 (2)
C31	0.045 (3)	0.025 (2)	0.025 (2)	0.0034 (19)	−0.012 (2)	−0.0065 (18)
C32	0.066 (3)	0.033 (3)	0.035 (3)	−0.001 (2)	−0.020 (2)	−0.007 (2)
C33	0.109 (5)	0.027 (3)	0.048 (3)	−0.006 (3)	−0.032 (3)	−0.002 (2)
C34	0.128 (5)	0.028 (3)	0.057 (4)	0.026 (3)	−0.049 (4)	−0.017 (3)
C35	0.087 (4)	0.042 (3)	0.052 (3)	0.034 (3)	−0.037 (3)	−0.025 (3)
C36	0.058 (3)	0.030 (2)	0.042 (3)	0.014 (2)	−0.021 (2)	−0.013 (2)
C37	0.048 (3)	0.022 (2)	0.027 (2)	0.0042 (19)	−0.009 (2)	−0.0047 (18)
C38	0.052 (3)	0.037 (3)	0.040 (3)	0.000 (2)	−0.014 (2)	−0.010 (2)
C39	0.069 (4)	0.046 (3)	0.065 (4)	−0.001 (3)	−0.033 (3)	−0.012 (3)
C40	0.099 (5)	0.045 (3)	0.066 (4)	−0.010 (3)	−0.046 (4)	−0.012 (3)
C41	0.109 (5)	0.042 (3)	0.042 (3)	0.002 (3)	−0.023 (3)	−0.019 (3)
C42	0.066 (3)	0.031 (2)	0.038 (3)	0.004 (2)	−0.016 (2)	−0.013 (2)
C43	0.124 (7)	0.398 (14)	0.106 (6)	−0.101 (8)	0.031 (5)	−0.185 (8)
O6	0.201 (7)	0.614 (17)	0.306 (11)	0.094 (10)	−0.050 (7)	−0.350 (12)
O7	0.069 (3)	0.088 (3)	0.056 (2)	0.026 (2)	−0.0123 (19)	−0.036 (2)
C44	0.095 (4)	0.066 (4)	0.050 (3)	0.001 (3)	−0.009 (3)	−0.031 (3)

### Geometric parameters (Å, °)

**Table:**

Pt1—P2^i^	2.3235 (9)	C17—H17A	0.9500
Pt1—P2	2.3236 (9)	C18—H18A	0.9500
Pt1—P1^i^	2.3399 (9)	C19—C20	1.396 (5)
Pt1—P1	2.3399 (9)	C19—C24	1.400 (5)
Pt1—O4	3.524 (3)	C20—C21	1.377 (5)
P1—C7	1.818 (3)	C20—H20A	0.9500
P1—C13	1.823 (3)	C21—C22	1.385 (6)
P1—C1	1.825 (3)	C21—H21A	0.9500
P2—C3	1.826 (3)	C22—C23	1.380 (6)
P2—C5	1.828 (3)	C22—H22A	0.9500
P2—C2	1.838 (3)	C23—C24	1.386 (5)
P3—O1	1.492 (2)	C23—H23A	0.9500
P3—C19	1.801 (4)	C24—H24A	0.9500
P3—C25	1.808 (3)	C25—C26	1.384 (5)
P3—C4	1.814 (3)	C25—C30	1.388 (5)
P4—O2	1.494 (3)	C26—C27	1.399 (5)
P4—C31	1.796 (4)	C26—H26A	0.9500
P4—C6	1.801 (3)	C27—C28	1.372 (5)
P4—C37	1.804 (4)	C27—H27A	0.9500
N1—O3	1.228 (4)	C28—C29	1.373 (5)
N1—O4	1.242 (4)	C28—H28A	0.9500
N1—O5	1.247 (4)	C29—C30	1.384 (5)
C1—C2	1.529 (4)	C29—H29A	0.9500
C1—H1B	0.9900	C30—H30A	0.9500
C1—H1C	0.9900	C31—C32	1.390 (5)
C2—H2B	0.9900	C31—C36	1.403 (5)
C2—H2C	0.9900	C32—C33	1.390 (6)
C3—C4	1.545 (4)	C32—H32A	0.9500
C3—H3A	0.9900	C33—C34	1.382 (7)
C3—H3B	0.9900	C33—H33A	0.9500
C4—H4A	0.9900	C34—C35	1.367 (6)
C4—H4B	0.9900	C34—H34A	0.9500
C5—C6	1.543 (4)	C35—C36	1.388 (5)
C5—H5A	0.9900	C35—H35A	0.9500
C5—H5B	0.9900	C36—H36A	0.9500
C6—H6A	0.9900	C37—C42	1.386 (5)
C6—H6B	0.9900	C37—C38	1.398 (5)
C7—C12	1.391 (4)	C38—C39	1.384 (6)
C7—C8	1.391 (5)	C38—H38A	0.9500
C8—C9	1.390 (5)	C39—C40	1.378 (7)
C8—H8A	0.9500	C39—H39A	0.9500
C9—C10	1.383 (5)	C40—C41	1.383 (7)
C9—H9A	0.9500	C40—H40A	0.9500
C10—C11	1.376 (5)	C41—C42	1.394 (6)
C10—H10A	0.9500	C41—H41A	0.9500
C11—C12	1.393 (5)	C42—H42A	0.9500
C11—H11A	0.9500	C43—O6	1.244 (8)
C12—H12A	0.9500	C43—H43A	0.9800
C13—C14	1.398 (5)	C43—H43B	0.9800
C13—C18	1.399 (5)	C43—H43C	0.9800
C14—C15	1.383 (5)	O6—H6C	0.8400
C14—H14A	0.9500	O7—C44	1.422 (5)
C15—C16	1.380 (5)	O7—H7A	0.8400
C15—H15A	0.9500	C44—H44A	0.9800
C16—C17	1.381 (5)	C44—H44B	0.9800
C16—H16A	0.9500	C44—H44C	0.9800
C17—C18	1.387 (5)		
			
P2^i^—Pt1—P2	180.0	C16—C15—C14	121.0 (4)
P2^i^—Pt1—P1^i^	83.40 (3)	C16—C15—H15A	119.5
P2—Pt1—P1^i^	96.60 (3)	C14—C15—H15A	119.5
P2^i^—Pt1—P1	96.60 (3)	C15—C16—C17	119.7 (4)
P2—Pt1—P1	83.40 (3)	C15—C16—H16A	120.2
P1^i^—Pt1—P1	180.0	C17—C16—H16A	120.2
P2^i^—Pt1—O4	80.23 (5)	C16—C17—C18	120.3 (4)
P2—Pt1—O4	99.77 (5)	C16—C17—H17A	119.8
P1^i^—Pt1—O4	75.64 (5)	C18—C17—H17A	119.8
P1—Pt1—O4	104.36 (5)	C17—C18—C13	120.2 (3)
C7—P1—C13	104.93 (16)	C17—C18—H18A	119.9
C7—P1—C1	105.46 (16)	C13—C18—H18A	119.9
C13—P1—C1	107.00 (16)	C20—C19—C24	118.4 (3)
C7—P1—Pt1	111.66 (12)	C20—C19—P3	118.1 (3)
C13—P1—Pt1	120.67 (11)	C24—C19—P3	123.5 (3)
C1—P1—Pt1	106.12 (11)	C21—C20—C19	121.7 (4)
C3—P2—C5	106.78 (15)	C21—C20—H20A	119.2
C3—P2—C2	106.38 (16)	C19—C20—H20A	119.2
C5—P2—C2	104.19 (16)	C20—C21—C22	118.9 (4)
C3—P2—Pt1	112.39 (11)	C20—C21—H21A	120.6
C5—P2—Pt1	117.14 (11)	C22—C21—H21A	120.6
C2—P2—Pt1	109.17 (11)	C23—C22—C21	121.0 (4)
O1—P3—C19	112.39 (16)	C23—C22—H22A	119.5
O1—P3—C25	112.26 (16)	C21—C22—H22A	119.5
C19—P3—C25	107.85 (16)	C22—C23—C24	119.9 (4)
O1—P3—C4	111.99 (16)	C22—C23—H23A	120.1
C19—P3—C4	106.38 (16)	C24—C23—H23A	120.1
C25—P3—C4	105.54 (16)	C23—C24—C19	120.2 (4)
O2—P4—C31	112.08 (17)	C23—C24—H24A	119.9
O2—P4—C6	111.78 (16)	C19—C24—H24A	119.9
C31—P4—C6	106.10 (17)	C26—C25—C30	118.7 (3)
O2—P4—C37	110.24 (17)	C26—C25—P3	118.5 (3)
C31—P4—C37	108.83 (17)	C30—C25—P3	122.8 (3)
C6—P4—C37	107.61 (17)	C25—C26—C27	120.2 (4)
O3—N1—O4	118.8 (4)	C25—C26—H26A	119.9
O3—N1—O5	121.3 (4)	C27—C26—H26A	119.9
O4—N1—O5	119.8 (4)	C28—C27—C26	120.4 (4)
N1—O4—Pt1	148.3 (3)	C28—C27—H27A	119.8
C2—C1—P1	108.3 (2)	C26—C27—H27A	119.8
C2—C1—H1B	110.0	C27—C28—C29	119.6 (4)
P1—C1—H1B	110.0	C27—C28—H28A	120.2
C2—C1—H1C	110.0	C29—C28—H28A	120.2
P1—C1—H1C	110.0	C28—C29—C30	120.5 (4)
H1B—C1—H1C	108.4	C28—C29—H29A	119.8
C1—C2—P2	110.6 (2)	C30—C29—H29A	119.8
C1—C2—H2B	109.5	C29—C30—C25	120.7 (4)
P2—C2—H2B	109.5	C29—C30—H30A	119.7
C1—C2—H2C	109.5	C25—C30—H30A	119.7
P2—C2—H2C	109.5	C32—C31—C36	119.1 (4)
H2B—C2—H2C	108.1	C32—C31—P4	118.4 (3)
C4—C3—P2	116.6 (2)	C36—C31—P4	122.5 (3)
C4—C3—H3A	108.1	C31—C32—C33	120.0 (5)
P2—C3—H3A	108.1	C31—C32—H32A	120.0
C4—C3—H3B	108.1	C33—C32—H32A	120.0
P2—C3—H3B	108.1	C34—C33—C32	120.3 (5)
H3A—C3—H3B	107.3	C34—C33—H33A	119.9
C3—C4—P3	109.4 (2)	C32—C33—H33A	119.9
C3—C4—H4A	109.8	C35—C34—C33	120.2 (5)
P3—C4—H4A	109.8	C35—C34—H34A	119.9
C3—C4—H4B	109.8	C33—C34—H34A	119.9
P3—C4—H4B	109.8	C34—C35—C36	120.4 (5)
H4A—C4—H4B	108.3	C34—C35—H35A	119.8
C6—C5—P2	111.1 (2)	C36—C35—H35A	119.8
C6—C5—H5A	109.4	C35—C36—C31	120.0 (4)
P2—C5—H5A	109.4	C35—C36—H36A	120.0
C6—C5—H5B	109.4	C31—C36—H36A	120.0
P2—C5—H5B	109.4	C42—C37—C38	118.8 (4)
H5A—C5—H5B	108.0	C42—C37—P4	119.2 (3)
C5—C6—P4	112.2 (2)	C38—C37—P4	122.0 (3)
C5—C6—H6A	109.2	C39—C38—C37	120.9 (4)
P4—C6—H6A	109.2	C39—C38—H38A	119.6
C5—C6—H6B	109.2	C37—C38—H38A	119.6
P4—C6—H6B	109.2	C40—C39—C38	120.0 (5)
H6A—C6—H6B	107.9	C40—C39—H39A	120.0
C12—C7—C8	119.7 (3)	C38—C39—H39A	120.0
C12—C7—P1	119.6 (3)	C39—C40—C41	119.8 (5)
C8—C7—P1	120.7 (3)	C39—C40—H40A	120.1
C9—C8—C7	119.7 (3)	C41—C40—H40A	120.1
C9—C8—H8A	120.1	C40—C41—C42	120.5 (5)
C7—C8—H8A	120.1	C40—C41—H41A	119.8
C10—C9—C8	120.3 (4)	C42—C41—H41A	119.8
C10—C9—H9A	119.8	C37—C42—C41	120.1 (4)
C8—C9—H9A	119.8	C37—C42—H42A	120.0
C11—C10—C9	120.2 (4)	C41—C42—H42A	120.0
C11—C10—H10A	119.9	O6—C43—H43A	109.5
C9—C10—H10A	119.9	O6—C43—H43B	109.5
C10—C11—C12	120.1 (3)	H43A—C43—H43B	109.5
C10—C11—H11A	119.9	O6—C43—H43C	109.5
C12—C11—H11A	119.9	H43A—C43—H43C	109.5
C7—C12—C11	119.9 (3)	H43B—C43—H43C	109.5
C7—C12—H12A	120.0	C43—O6—H6C	109.5
C11—C12—H12A	120.0	C44—O7—H7A	109.5
C14—C13—C18	119.1 (3)	O7—C44—H44A	109.5
C14—C13—P1	120.8 (3)	O7—C44—H44B	109.5
C18—C13—P1	120.0 (3)	H44A—C44—H44B	109.5
C15—C14—C13	119.7 (3)	O7—C44—H44C	109.5
C15—C14—H14A	120.1	H44A—C44—H44C	109.5
C13—C14—H14A	120.1	H44B—C44—H44C	109.5

Symmetry codes: (i) −*x*, −*y*, −*z*.

### Hydrogen-bond geometry (Å, °)

**Table:**

*D*—H···*A*	*D*—H	H···*A*	*D*···*A*	*D*—H···*A*
O6—H6C···O2	0.84	2.32	3.160 (9)	174
O7—H7A···O2	0.84	1.95	2.777 (4)	170
C12—H12A···O4	0.95	2.29	3.132 (4)	147
C6—H6B···O4	0.99	2.53	3.294 (4)	134
C1—H1B···O5^ii^	0.99	2.42	3.289 (5)	146
C1—H1C···O5^iii^	0.99	2.44	3.399 (5)	162
C8—H8A···O3^ii^	0.95	2.58	3.376 (5)	142
C14—H14A···O4^iii^	0.95	2.56	3.317 (5)	137
C27—H27A···O6^iv^	0.95	2.50	3.338 (11)	147
C28—H28A···O2^iv^	0.95	2.60	3.385 (5)	141

Symmetry codes: (ii) *x*+1, *y*, *z*; (iii) −*x*, −*y*, −*z*; (iv) −*x*+1, −*y*, −*z*−1.
